# Exogenous Paclobutrazol Reinforces the Antioxidant and Antimicrobial Properties of Lavender *(Lavandula officinalis* L.) Oil through Modulating Its Composition of Oxygenated Terpenes

**DOI:** 10.3390/plants11121607

**Published:** 2022-06-19

**Authors:** Salwa M. El-Sayed, Karim. M. Hassan, Ahmed. N. Abdelhamid, Eman E. Yousef, Yasmin M. R. Abdellatif, Samah H. Abu-Hussien, Mohamed A. Nasser, Walaa. A. Elshalakany, Doaa Bahaa Eldin Darwish, Awatif M. Abdulmajeed, Nadiyah M. Alabdallah, Salem Mesfir Al-Qahtani, Nadi Awad Al-Harbi, Eldessoky S. Dessoky, Hatem Ashour, Mohamed F. M. Ibrahim

**Affiliations:** 1 Department of Biochemistry, Faculty of Agriculture, Ain Shams University, Cairo 11566, Egypt; salwa_sedeek@agr.asu.edu.eg (S.M.E.-S.); walaa_elshalakani@agr.asu.edu.eg (W.A.E.); 2 Department of Horticulture, Faculty of Agriculture, Ain Shams University, Cairo 11566, Egypt; kareem_hassan@agr.asu.edu.eg (K.M.H.); ahmed_nazmy@agr.asu.edu.eg (A.N.A.); mohamed_21884@agr.asu.edu.eg (M.A.N.); 3 Department of Food Science and Technology, Faculty of Agriculture, Ain Shams University, Cairo 11566, Egypt; emanyousef@agr.asu.edu.eg; 4 Department of Agricultural Botany, Faculty of Agriculture, Ain Shams University, Cairo 11566, Egypt; dr_yasminmarzouk@agr.asu.edu.eg (Y.M.R.A.); hatem_ashour@agr.asu.edu.eg (H.A.); 5 Department of Microbiology, Faculty of Agriculture, Ain Shams University, Cairo 11566, Egypt; samah_hashem1@agr.asu.edu.eg; 6 Botany Department, Faculty of Science, Mansoura University, Mansoura 35511, Egypt; ddarwish@ut.edu.sa; 7 Biology Department, Faculty of Science, University of Tabuk, Umluj 46429, Saudi Arabia; awabdulmajeed@ut.edu.sa; 8 Department of Biology, College of Science, Imam Abdulrahman Bin Faisal University, P.O. Box 1982, Dammam 31441, Saudi Arabia; nmalabdallah@iau.edu.sa; 9 Biology Department, University College of Tayma, University of Tabuk, P.O. Box 741, Tabuk 47512, Saudi Arabia; salghtani@ut.edu.sa (S.M.A.-Q.); nalharbi@ut.edu.sa (N.A.A.-H.); 10 Department of Biology, College of Science, Taif University, P.O. Box 11099, Taif 21944, Saudi Arabia; es.dessouky@tu.edu.sa

**Keywords:** *Lavandula officinalis* L., Gas chromatography-mass spectrometry (GC-MS), chemical composition, monoterpene and sesquiterpene

## Abstract

Plant growth regulators can affect the primary and secondary metabolites of various plant species. However, the effect of paclobutrazol (PBZ) on the composition of lavender oil, especially related to the terpenoid pathway, is still unclear in literatures. In this study, the effect of PBZ as a foliar spray (0.200, 400 and 600 ppm) on the vegetative growth, phytochemical content, and both antioxidant and antimicrobial properties of lavender oil were investigated. The results indicated that all examined PBZ treatments led to a significant (*p* ≤ 0.05) decrease in growth parameters compared to the untreated plants. Meanwhile, the yield of essential oil was significantly decreased by the treatment of PBZ at 200 ppm compared to the control. In contrast, applied-PBZ significantly enhanced the chlorophyll content and displayed a marked change in the composition of the essential oil. This change included an obvious and significant increase in 3-carene, eucalyptol, γ–terpinene, α-pinocarvone, caryophyllene, β-vetivenene, β-santalol, ledol, geranyl isovalerate, farnesol, caryophyllene oxide, and phytol percentage. Generally, the highest significant values were achieved by the treatment of 400 ppm compared to the other treatments. Furthermore, this treatment showed the highest free radical scavenging activity against DPPH (1,1-diphenyl-2-picrylhydrazyl) by 13% over the control. Additionally, to determine the antimicrobial activities of the extracted oil, each treatment was examined against two strains of Gram positive bacteria (*S. aureus* and *B. cereus*), two strains of Gram negative bacteria (*S. enteritidis* and *E. coli*), and two fungal species (*C. albicans* and *A. niger*) represent the yeast modal and filamentous fungus, respectively. The findings demonstrated that all examined species were more sensitive to the oil that was extracted from lavender plants, treated with 400 ppm PBZ, compared to the other concentrations.

## 1. Introduction

Lavender is the common name of the plant genus *Lavandula* (family, *Lamiaceae*), which comprises several plant species with economic, aromatic, and medicinal importance [1]. The extracted phytochemicals of genus *Lavandula* are widely used for cosmetics [2], food and flavor industries [3], and pharmaceutical products [1,4,5]. For centuries, lavender essential oils were used to cure pain, parasite infections, burns, insect bites, cramps, and muscular spasms [2,3,6,7]. Furthermore, it has been found that these essential oils possess antibacterial [8,9,10], anti-inflammatory [11,12], anticancer [13], and antioxidant properties [9,14,15].

Generally, essential oils are a mixture of bioactive molecules that have antioxidant and antimicrobial activities, including monoterpenes, sesquiterpenes, and phenylpropanoids [16,17,18]. Monoterpenes have been found to have antibacterial, antifungal, and cytotoxic properties in cancer cell lines [19,20]. In general, essential oil ingredients can be divided into two parts: hydrocarbons (monoterpenes, sesquiterpenes, and diterpenes), and oxygenated substances (oxygenated terpenoids) [21]. Oxygenated terpenoids, such as oxygenated monoterpenes and oxygenated sesquiterpenes, have stronger antimicrobial activity than other terpenoids [17,18]. The strongest antimicrobial activity of oxygenated monoterpenes, such as α-terpineol, linalool, and eucalyptol (1,8-cineole) was attributed to the presence of hydroxyl groups (-OH), which may also be responsible for the interaction with intracellular components of microorganisms [17]. Additionally, as β-caryophyllene-containing essential oils have antibacterial properties against both Gram-positive and Gram-negative bacteria [17]. Conversely, monoterpene hydrocarbons (e.g., α-pinene, camphene, myrcene, α-terpinene, and p-cymene) show limited antibacterial activity [22]. On the other hand, monoterpenes are considered the most potent compounds, which are responsible for the antioxidant effects of essential oils [23]. It has been found that α-pinene and α-phellandrene react quickly with peroxyl radicals, leading to an early termination of oxidative chain reactions and a reduction in the quantity of reactive radicals [24,25]. Furthermore, the presence of significantly activated methylene groups or tertiary allylic alcohol, in volatile substances such as eucalyptol, could result in considerable antioxidant capabilities [26]. These substances can contribute an electron to radicals such as H_2_O_2_ and, then, reduce them to non-radical forms such as H_2_O and O_2_ [14].

Paclobutrazol (PBZ) is a plant growth regulator that serves as a retardant to cell elongation without an effect on the rate of cell division [27]. It can temporarily restrict the gibberellins (GA_s_) biosynthesis by preventing the oxidation of ent-kaurene to ent-kaurenoic acid through inactivating cytochrome P-450-dependent oxygenases [28,29]. When GA_s_ biosynthesis is restricted, more precursors in the terpenoid pathway are accumulated and shunted to induce abscisic acid (ABA) biosynthesis [27,30]. Due to their antioxidant and antimicrobial activities, accumulation of terpenes can stimulate plant tolerance to various biotic and abiotic stresses [17,18]. Moreover, PBZ belongs to the triazole family, which is widely used as fungicide in agriculture [31]. Applied-PBZ has been found to have several benefits in the previous studies. In this context, exogenous PBZ enhanced the leaf water potential of young apple trees under drought stress [29], as well as pomegranate tolerance to freezing stress [32]. Applied PBZ also improved sesame yield by increasing dry matter accumulation and reducing seed shattering under rainfed conditions [33]. Meanwhile, applied PBZ induced disease resistance against *Alternaria* leaf spot in Faba beans [34]. On the other hand, it is well documented that exogenous applied PBZ can induce ABA synthesis [27]. This increase in ABA can serve as a signaling molecule under various abiotic stresses, including heavy metals [35], salinity [36], drought [37], and chilling [38]. Furthermore, ABA can induce stomatal closure, which is considered an important component of plant defense against abiotic and biotic stress [39].

This study was conducted to (i) evaluate the effect of exogenous PBZ on changing the composition of lavender (*Lavandula officinalis* L.) essential oil using GC- Mass chromatographic technique (ii) and to further understand how far these modifications in the composition of essential oil can affect the antioxidant and antimicrobial activities *in vitro*.

## 2. Results

### 2.1. Effect of PBZ on the Vegetative Growth of Lavender Plants

**Figure 1:**
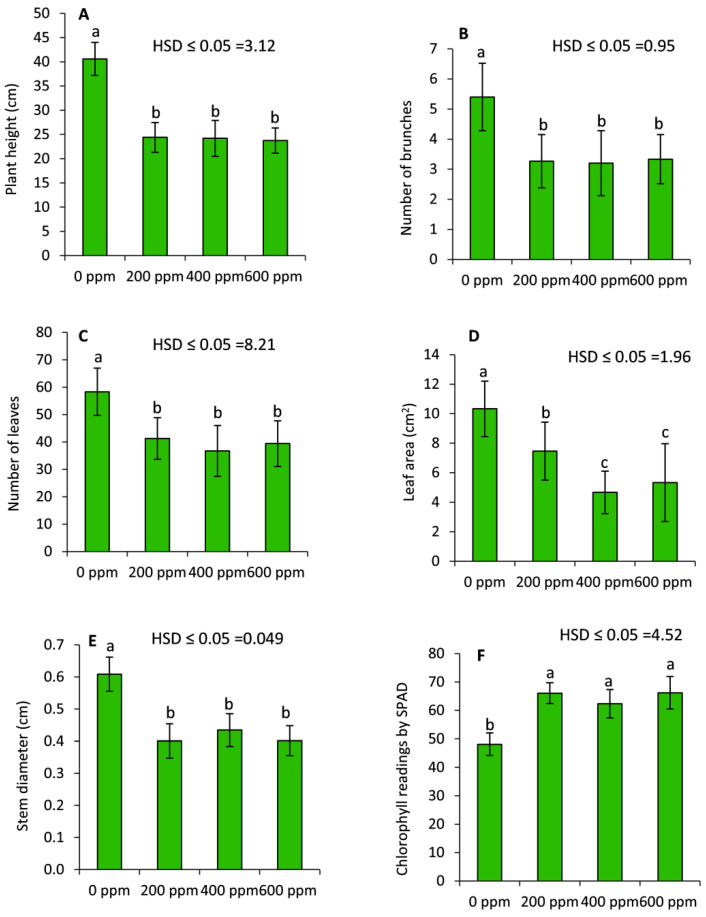
Effect of paclobutrazole (PBZ) on the vegetative growth of lavender plants and chlorophyll readings by SPAD. Values are the averages of 15 plants ± SD. Different letters indicate significant differences according to Tukey’s Studentized Range (HSD) Test (*p* < 0.05).

Data presented in Figure 1 show that plants treated by PBZ demonstrated a significant (*p* ≤ 0.05) decrease in the vegetative growth, including plant height, number of brunches, number of leaves, leaf area, and stem diameter compared to the untreated plants. Conversely, chlorophyll readings by SPAD exhibited an obvious and significant increase in all PBZ-treatments compared to the untreated plants. Generally, except in the leaf area, no significant differences were detected between the various examined concentrations of PBZ.

### 2.2. Effect of PBZ on the Yield of Lavender Essential Oil

**Figure 2:**
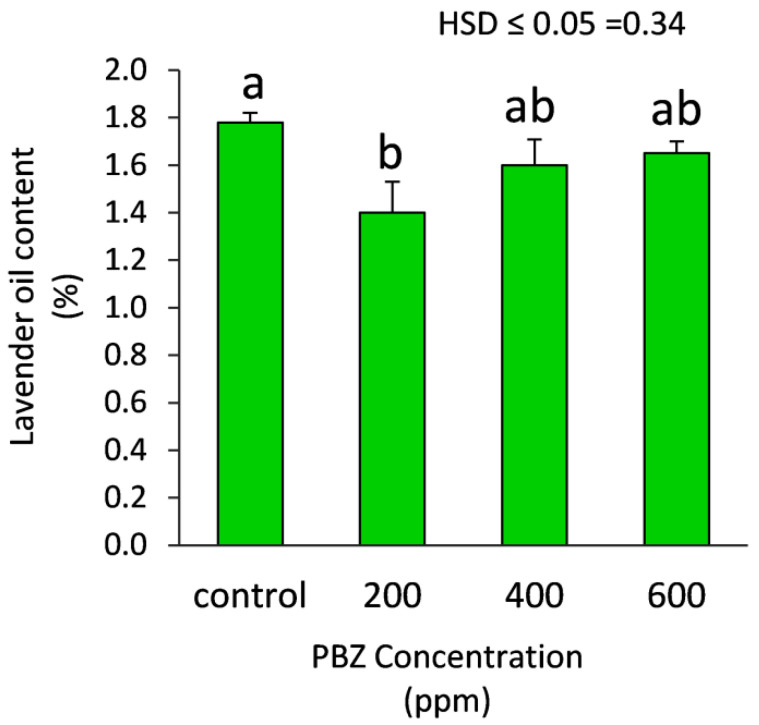
Effect of paclobutrazole (PBZ) on the yield of lavender essential oil (%) in the dried vegetative parts (leaves and stem). Values are the averages of 3 replicates ± SD. Different letters indicate significant differences, according to Tukey’s Studentized Range (HSD) Test (*p* < 0.05).

Data presented in Figure 2 show that plants treated with PBZ at 200 ppm demonstrated an obvious and significant (*p* ≤ 0.05) decrease in the yield of essential oil compared to the untreated plants. Meanwhile, the treatments of PBZ at 400 and 600 ppm did not significantly affect the yield of essential oil compared to the untreated plants.

### 2.3. Effect of PBZ on Lavender Essential Oil Composition

**Figure 3:**
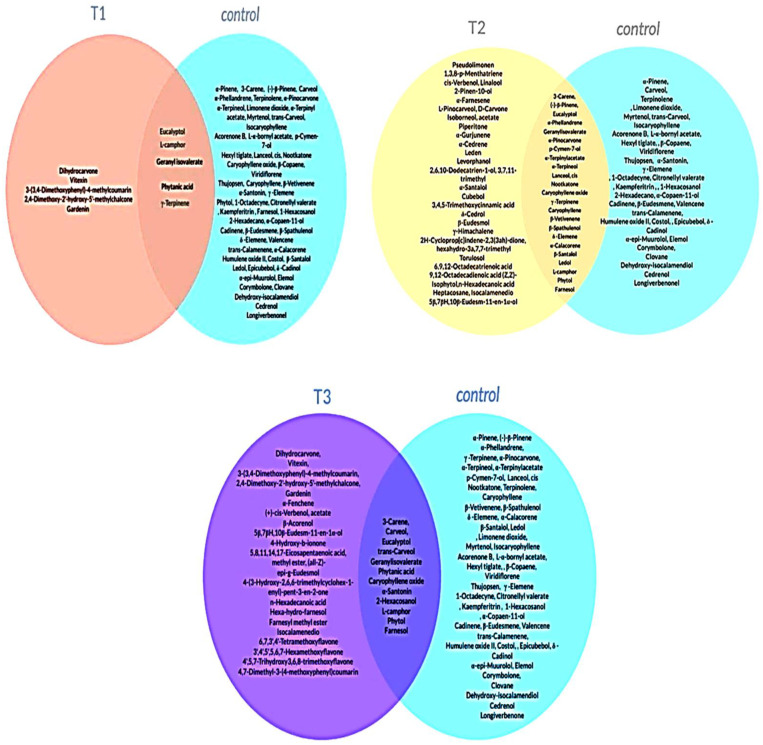
Comparative volatile compositions of essential oil extracted from untreated and treated lavender plants with paclobutrazole; PBZ: (**T1**) Venn diagram of volatile compounds of lavender oils extracted from lavender plants, untreated (control) and treated, with PBZ at 200 ppm (T1), (**T2**) Venn diagram of volatile compounds of lavender oils extracted from lavender plants, untreated (control) and treated, with PBZ at 400 ppm (T2), and (**T3**) Venn diagram of volatile compounds of lavender oils extracted from lavender plants, untreated (control) and treated, with 600 ppm paclobutrazole PBZ (T3).

According to the data obtained by GC-MS analysis (Supplementary Table S1), about 108 different compounds were detected in the essential oil extracted from PBZ-treated and untreated plants. Generally, both oxygenated monoterpenes (Eucalyptol and L-camphor) represent the highest percentages (40–45%) of the total found compounds in the extracted oil from PBZ-treated and untreated plants. However, applied-PBZ, at different concentrations, revealed several differences in the composition of essential oil compared to the untreated plants (Figure 3A–C). The comparative study of volatile compositions demonstrated that the oxygenated monoterpene (dihydrocarvone) and sesquiterpene (caryophyllene oxide) were common in the PBZ-treated plants and absent in the control.

### 2.4. Effect of PBZ on the Principal Component Analysis (PCA) of Lavender Essential Oil

**Figure 4:**
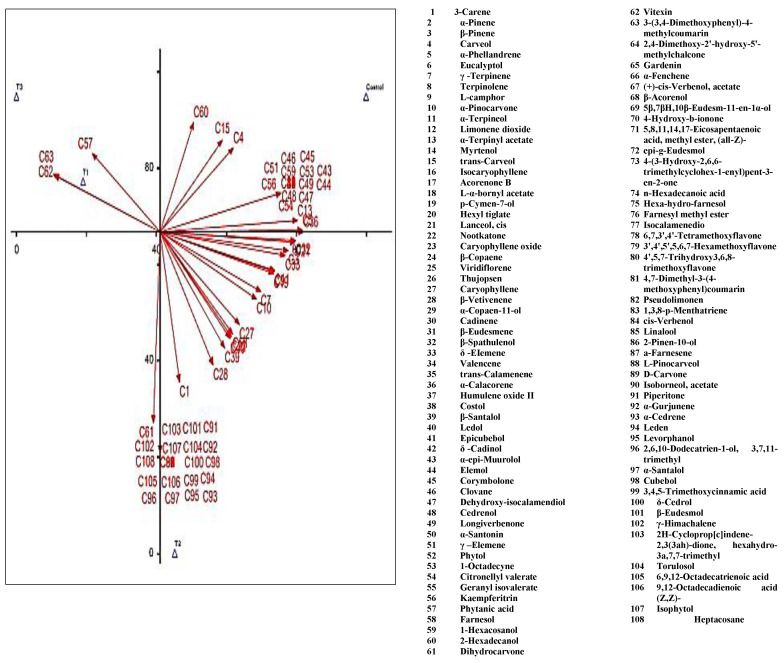
Principal component analysis (PCA) of the essential oil extracted from untreated and treated lavender plants with paclobutrazole.

To further understand the differences between the compositions of essential oil extracted from PBZ-treated and untreated plants, principal component analysis was conducted on the common volatiles (Figure 4). The results indicated that the oil of the untreated plant exhibited high scores on positive PC, where the loadings of characteristic volatile compounds were high, including α-terpinyl acetate, valencene, δ-cadinol, α-epi-Muurolol, elemol, corymbolone, clovane, dehydroxy-isocalamendiol, cedrenol, longiverbenone, and γ-elemene. On the other hand, the treatment of 400 ppm PBZ showed high scores in positive PC, which contained high loadings of compounds, including 3-carene, β-pinene, γ –terpinene, α-terpineol, eucalyptol, p-cymen-7-ol, β-santalol, β-spathulenol, δ –eiemene, α-pinocarvone, α-santonin, and caryophyllene oxide. In contrast, the treatment of 600 ppm PBZ exhibited high scores in negative PC, which contained high loadings of two compounds, including dihydrocarvone and vitexin. Most of these compounds were absent in the oil that was extracted from plants treated by 200 ppm PBZ.

### 2.5. Effect of PBZ on the Quantity of Monoterpene and Sesquiterpene Constituents

To better focus on the compounds of highly efficient antioxidative and antimicrobial activities, the quantity of monoterpene and sesquiterpene constituents was identified using GC-MS analysis (Table 1). These compounds can be categorized into four classes, including monoterpene hydrocarbons, oxygenated monoterpenes, sesquiterpene hydrocarbons, and oxygenated sesquiterpenes. The results revealed that lavender oil extracted from plants treated with 400 ppm PBZ was higher in the oxygenated monoterpene (54%), sesquiterpene hydrocarbons (4.41%), and oxygenated sesquiterpene (5.68%) than the untreated plants, which recorded 49.41, 0.69, and 1.93% in these biochemical classes, respectively. In contrast, despite the treatment of 200 ppm PBZ, it showed a decrease in the total quantity of oxygenated monoterpenes, but it displayed an obvious increase in Eucalyptol compared to the untreated plants. On the other hand, the treatment of 600 ppm PBZ exhibited a substantial increase in a single compound of the oxygenated sesquiterpenes (caryophyllene oxide) compared to the untreated plants. These results may imply that the antioxidant and antimicrobial activities could be attributed to the presence of specific compounds, regardless the total quantity of the rest of the compounds that belong to the same biochemical class.

### 2.6. Effect of PBZ on the Antioxidant Activity

**Figure 5:**
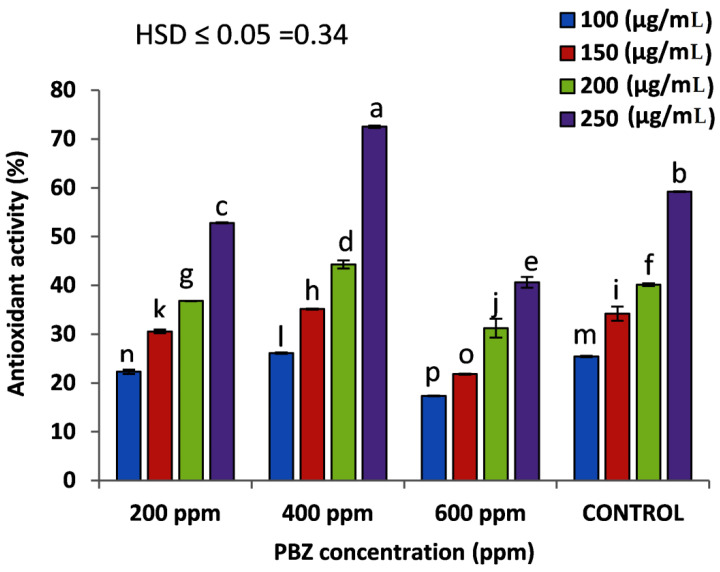
DPPH scavenging activity of different concentrations (100, 150, 200, and 250 µg/mL) of the essential oil extracted from lavender (*Lavender officinalis*) plants, treated and untreated with paclobutrazole; PBZ. Values are the averages of 3 replicates ± SD. Different letters indicate significant differences, according to Tukey’s Studentized Range (HSD) Test (*p* < 0.05).

To evaluate the total antioxidant capacity of the extracted essential oil from different PBZ-treated and untreated plants, free radical scavenging activity against DPPH was used in vitro (Figure 5). The results indicated that the treatment of 400 ppm PBZ, with the dose of 250 µg/mL of the extracted oil, revealed the highest significant (*p* ≤ 0.05) antioxidant capacity (72.54%) compared to the same dose of the untreated plants (59.19). Generally, the antioxidant capacity was significantly increased in parallel with increasing the used dose of extracted oil from 100–250 µg/mL in all studied treatments. 

### 2.7. Effect of PBZ on the Antimicrobial Activity

**Figure 6:**
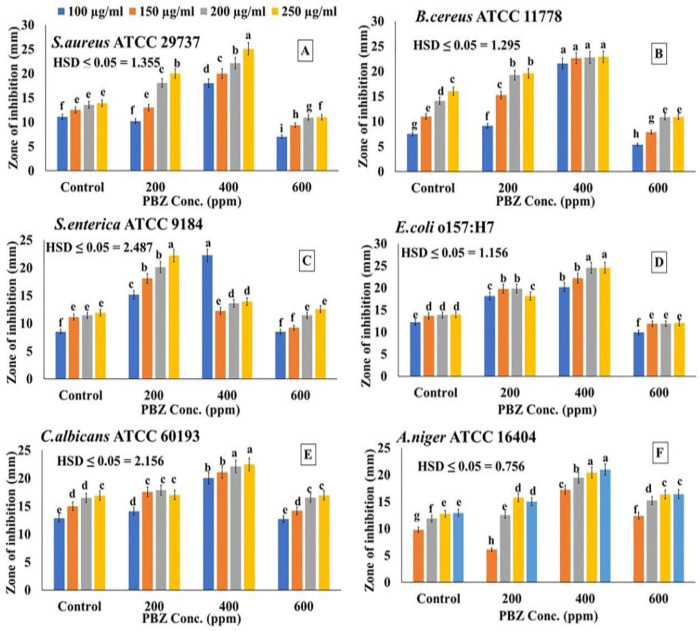
The antibacterial and antifungal activities of the essential oil extracted from lavender (*Lavender officinalis*) plants, treated by paclobutrazole (PBZ) at 0, 200, 400, and 600 ppm, against *S. aureus* (**A**), *B. cereus* (**B**), *S. enterica* (**C**), *E. coli* (**D**), *C. albicans* (**E**), and *A. niger* (**F**). Values are the averages of 3 replicates ± SD. Different letters indicate significant differences, according to Tukey’s Studentized Range (HSD) Test (*p* < 0.05).

In addition to the antioxidant capacity, the antibacterial and antifungal activities of the extracted oil, from PBZ-treated and untreated plants, were investigated (Figure 6). Four different concentrations (100, 150, 200, and 250 µg /mL) of each extract (50 µL per disc) were examined for their positive antimicrobial activity and increasing the inhibition zone diameter (mm). Oils extracted from lavender treated plants with 400 ppm PBZ showed strong and significant (*p* ≤ 0.05) antibacterial activity against two pathogenic Gram-negative (*E. coli* and *S. enteritidis*) and Gram-positive (B. Subtilis and S. aureus) bacterial strains compared to the oil extracted from the other treatments. A simillar trend was also observed with respect to the antifungal activities against A. niger and C. albicans. This promising antifungal activity was also evidenced by increasing the inhibition zone diameter (mm) of fungal growth *in vitro.*

## 3. Discussion

In this study, lavender plants treated with PBZ revealed a significant inhibition in growth parameters compared to the untreated plants. It is well documented that PBZ, as a plant growth retardant, can affect the vegetative growth by mediating a number of changes in the levels of other plant growth regulators, including auxins, gibberellins, abscisic acid, and cytokinins [27,30,40]. It can temporarily restrict the gibberellins biosynthesis by preventing the oxidation of ent-kaurene to ent-kaurenoic acid through inactivating cytochrome P-450-dependent oxygenases [28,29]. This response can stimulate the isoprenoid pathway, leading to ABA synthesis, which is considered to be the main plant growth inhibitor in higher plants [27,30]. In contrast, applied PBZ led to an obvious and significant increase in the readings of chlorophyll by SPAD. This effect could be attributed to the ability of PBZ to increase the phytol, which is involved in the carbon skeleton of chlorophyll molecule [27]. Moreover, the total yield of lavender essential oil was negatively, and significantly, affected by different PBZ treatments. This response could be due to the inhibition of vegetative growth parameters, where the leaves and stem are the major parts used for oil extraction in this study.

Lavender oil is considered a complex mixture of many secondary metabolites, including terpenes, alcohols, aldehydes, and phenols [41]. This composition can be affected by several factors, i.e., plant age, geography, climatic conditions, plant organ selection, harvest season, and the extraction method [42]. Furthermore, exogenous PBZ has been found to stimulate the terpenoid pathway, leading to the accumulation of several terpenes and restrict the biosynthesis of GA_3_ [27,43]. In this study, lavender plants treated with 200 ppm PBZ demonstrated an increase in eucalyptol, geranyl isovalerate, and phytanic acid compared to the untreated plants. Meanwhile, the treatment of 400 ppm PBZ showed considerable accumulation in 3-carene, eucalyptol, γ-terpinene, α-pinocarvone, caryophyllene, β-vetivenene, β-santalol, ledol, geranyl isovalerate, farnesol, caryophyllene oxide, and phytol compared to the untreated plants. On the other hand, the treatment of 600 ppm PBZ displayed an obvious increase in caryophyllene oxide, α-santonin, geranyl isovalerate, phytanic acid, and phytol compared to the untreated plants. These findings imply that applied PBZ can affect the profile of oil constituents, which related to the monoterpenes and sesquiterpenes in both their forms (oxygenated compounds or hydrocarbons). These secondary metabolites possess a large scale of defensive effects against insects, viruses, bacteria, and fungi [14,18,44,45,46]. Furthermore, this protective effect can be extended to enhance plant tolerance against various abiotic stresses, due to the antioxidant properties of these compounds [9,17,26,47]. In this study, the treatment of 400 ppm PBZ, followed by the control, revealed a greater antioxidant capacity compared to the other treatments. These results could be attributed to increasing the percentage of terpenes (Table 1). Terpenes can break the chain and react with the lipid peroxyl radicals, leading to formation stable molecules [47]. Besides, in this study, the treatment of 400 ppm showed the presence of linolool (oxygenated monoterpene), which has strong antioxidant power, due to its hydrogen atom donation and electron removal, from the macromolecules leading to prevent the oxidative damage [48]. Similarly, several previous studies reported that the terpenes and terpenoids of essential oils can contribute to the antioxidant activity, such as α-terpinene, β-terpinene and β-terpinolene [49], 1,8-cineole (Eucalyptol) [50], menthone and isomenthone [51], thymol, eugenol, and linalool [52]. Generally, earlier studies identified a strong link between the chemical content of essential oils and their antioxidant activity, particularly when molecules possess hydroxyl functionalities [53]. In this context, it was reported that essential oils that are rich in oxygenated monoterpenes possess high antioxidant potency [54]. This effect was in harmony with the findings obtained in this study.

Many researchers found that most of the plants that contain high levels of essential oils have inhibitory potentials against pathogenic microorganisms. Therefore, these oils can be used as antimicrobial compound drugs. The inhibitory potentials of lavender essential oil, against bacterial and fungal pathogens, were investigated in this study. The results showed antibacterial inhibitory effects against *S. aureus* ATCC 29737, *B. cereus* ATCC 11778, *S. entertidis* ATCC, *E. coli* o157:H7, *C. albicans* ATCC 60193, and *A. niger* ATCC 16404. The antibacterial action of lavender essential oil is mostly correlated with the presence of active components, such as monoterpenes, sesquiterpenes, and their derivatives. Recent research has found that various essential oils, including lavender and basil, have antibacterial properties against Gram-positive bacteria, such as *Staphylococcus aureus* and *Bacillus* species, Gram-negative bacteria, such as *Escherichia coli* and *Shigella flexneri*, as well as the pathogenic fungi, such as *Candida albicans* [55,56,57]. Furthermore, it has been found that hydrodistilled coriander essential oil has a potent antifungal activity against *C. albicans* [57]. This essential oil of coriander can cause a synergistic antifungal activity against *Candida species* and potential synergism with amphotericin B [58]. Additionally, Rahman, et al. [59] found that the essential oil extracted from the leaves of *Piper chaba Hunter* displayed potent antifungal activity against *Fusarium oxysporum*, *Phytophthora capsici, Colletotrichum capsici, Fusarium solani*, and *Rhizoctonia solani*. This influence was attributed to the activity to α-humulene, caryophyllene oxide, viridiflorol, globulol, β-selinene, spathulenol, (E)-nerolidol, linalool, 3-pentanol, and *p*-cymene that were present in the oil. Several investigations proposed that the mechanism of action of monoterpenes and its derivatives affect the cell membrane permeability, based on their ability to stop cell wall synthesis and damage the cytoplasmic membrane, resulting in lysis and leakage of intracellular components [60]. Additionally, our results were similar to those reported elsewhere [61], which showed that essential oil derived from lavender displayed significant antibacterial activity against *Bacillus subtilis* and *Aspergillus niger*. In the present work, the findings were in agreement with previous studies that linked essential oil’s antibacterial potency to the presence of a considerable amount of camphor [62], confirming that oxygenated monoterpenes had antibacterial properties against a variety of bacteria.

The mechanism of action of camphor can enter cell walls and the cytoplasmic membrane, causing polysaccharide structure, fatty acid, and phospholipid permeability [63]. The mechanism of action of oxygenated monoterpenes, such as 1,8-cineole (eucalyptol), is most likely due to their ability to form hydrogen bonds, which defines their activity against Gram positive bacteria [64]. Since camphor and eucalyptol are the most important components of the essential oils in this study (Table 1), it is possible that they are responsible for the reported antimicrobial properties of essential oils under the circumstances of this study. The molecular interaction of the functional groups of the components with the bacteria wall [65], which results in deep lesions, may also explain the strong antibacterial activity of lavender essential oil extracted from plants treated with 400 ppm PBZ.

## 4. Material and Methods

### 4.1. Plant Material, Treatments and Growth Parameters

**Figure 7:**
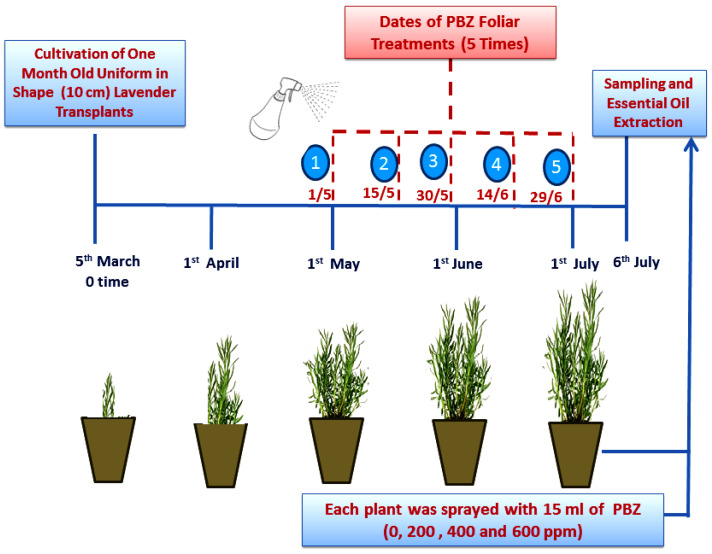
The timeline infographic for the treatments of paclobutrazol (PBZ), as a foliar application at 0, 200, 400, and 600 ppm and the sampling date, to extract the essential oil of lavender (*Lavandula officinalis* L.) plants.

One month old, healthy and uniform in shape, lavender (*Lavandula officinalis* L.) transplants (10 cm, length) were purchased from a private nursery, Giza, Egypt. Each single transplant was cultivated in the first week of March (2021) in a plastic pot (35 cm diameter) filled with peat moss and sand (1:1). The irrigation was regularly done 2–3 times a week after calculating the decrease in water-holding capacity using the weight method. Fertilization was also done using a half-strength Hoagland’s nutrient solution (one time every 10 days). After 2 months of cultivation, all pots (60 pots) were divided into 4 groups, in the first week of May, to apply the foliar applications of α-tert-Butyl-β-(4-chlorobenzyl)-1H-1,2,4-triazole-1-ethanol (paclobutrazol; PBZ; Zeneca ICI Agrochemical Ltd., Mumbai, India) at 0 (distilled water as a control), 200, 400, and 600 ppm. Each group of plants (15 pots) was sprayed five times with 15 mL of a specific concentration of PBZ solutions, as shown in Figure 7. In the first week of July, plants were gathered to determine the growth parameters and record the chlorophyll content, based on the chlorophyll SPAD readings, using a digital chlorophyll meter (Minolta SPAD-502, Japan). The experimental layout was of complete randomized design (CRD) with 3 replicates. All the experimental pots were distributed as follows:

4 PBZ treatments × 5 pots × 3 replicate = 60 plants

### 4.2. Extraction and Determination of Lavender Essential Oil Content

Steam distillation was used to isolate the essential oil of dried vegetative parts of the lavender plant, the leaves and stem (untreated and treated with PBZ), using a Clevenger glass apparatus, in which 100 g of the dried whole lavender plant was extracted in the steam distillation apparatus for 3 h. Lavender oil was isolated from the remaining water and stored in dark glass vials at 4 °C until the active compounds were separated and analyzed by Gas chromatography—mass (GC-MS). The oil yield was calculated as grams of oil per 100 g of dry whole lavender plant. 

### 4.3. Gas Chromatography-Mass Spectrometry (GC-MS) Analysis

After evaporation, the extracted oil residue was dissolved with 3 mL ethyl acetate, and the extracted oil residue was dissolved with 3 mL ethyl acetate then 1 mL transferred to GC vial for GC/MS analysis. Gas chromatography—mass was used for the analysis of various components of lavender volatile oil that were present in modest quantities, in addition to the analysis of the main components of lavender essential oil. The identification of components was based on a comparison of their mass spectra and retention time with those of the authentic compounds and by computer matching with NIST and WILEY library, as well as by comparison of the fragmentation pattern of the mass spectral data with those reported in the literature. The analysis was carried out using a GC (Agilent Technologies 7890A) interfaced with a mass-selective detector (MSD, Agilent 7000) and equipped with a polar Agilent HP-5ms (5%-phenyl methyl poly siloxane) capillary column (30 m × 0.25 mm i.d. and 0.25 μm film thickness). The carrier gas was helium, with the linear velocity of 1 mL/min. The injector and detector temperatures were 200 °C and 250 °C, respectively, while volume injected 1 μL of the sample. The MS operating parameters were as follows: ionization potential 70 eV, interface temperature 250 °C, and acquisition mass range 50–800 [66].

### 4.4. DPPH Free Radical Scavenging Activity

The capacity of essential lavender oil to scavenge the DPPH (1,1-diphenyl-2-picrylhydrazyl) radical was determined, according to the method described by Gargouri et al. [67]. In the DPPH method, 500 µL of freshly prepared DPPH solution (50 mM in absolute ethanol) was mixed with 1 mL of lavender oil (100, 150, 200, and 250 µL/L) and left in the dark for 30 min. Then, the absorbance of the mixture was recorded at 517 nm. The capability to scavenge the DPPH radical (% inhibition) was calculated using the following equation:

(1) $$\%\;\mathrm{inhibition}\;=\left[\frac{A_{c}-A_{t}}{A_{c}}\right]\;\times \;100$$

where, A_c_ is the absorbance of reaction without the sample (control) and A_t_ is the absorbance of the test samples.

### 4.5. Pathogenic Microbial Strains

Six pathogenic microbial strains, including *S. aureus* ATCC 29737, *B. cereus* ATCC 11778, *S. enterica* ATCC 9184, *C. albicans* ATCC 60193, and *A. niger* ATCC 16404, were collected from Microbiological Resource Center (MERCIN) at Faculty of Agriculture, Ain Shams University, Cairo, Egypt, and *E. coli* o157:H7 was purchased from Microbiological Laboratory of Animal Health Institute, Cairo, Egypt. All tested microorganisms were cultured on Mueller Hinton Agar (MHA), followed by culturing on Tryptic Soy Broth (TSB), and incubated at 37 °C for 24 h. All cultures were then kept at 4 °C for further experiments. A loopful of each studied pathogenic microbial strain (10^6^ CFU/mL) was determined by the plate count method and inoculated into a 200 mL Erlynemyer flask containing 100 mL of Tryptic Soy Broth, and it was incubated at 37 °C, under shaking at 150 4pm, for 24 h [68].

### 4.6. Antimicrobial Activity of Lavendar Essential Oil Using Well Diffusion Method

The inhibitory activity of lavender essential oil was tested against six pathogenic microorganisms, including four bacterial strains (*S. aureus* ATCC 29737, *B. cereus* ATCC 11778, *S. enterica* ATCC 9184, *E. coli* o157:H7) and two fungal strains (*C. albicans* ATCC 60193 and *A. niger* ATCC 16404). Different lavender essential oil concentrations (100, 150, 200, and 250 µg/mL) were prepared by dissolving them in 100% dimethyl sulfoxide (DMSO). Briefly, one milliliter of all previous pathogens’ inocula were spread onto sterile MHA plates. For wells preparation, Agar plates were holed using a sterile 7 mm diameter cork-borer. Each well was filled with 100 µL of lavender essential oil concentrations, individually. All plates were kept at room temperature for 1 h, followed by incubation at 37 °C for 24 h, according to the CLSI [69] method. DMSO was served as the negative control. All experiments were carried out in triplicates. Antimicrobial activity was determined by measuring the diameter of clear zones in millimeters.

### 4.7. Statistical Analysis

One way ANOVA procedure was followed using SAS [70] software. Means ± SD were calculated from three replicates and Tukey’s Studentized Range (HSD) Test (*p* < 0.05) was used to determine the significant differences between means.

## 5. Conclusions

**Figure 8:**
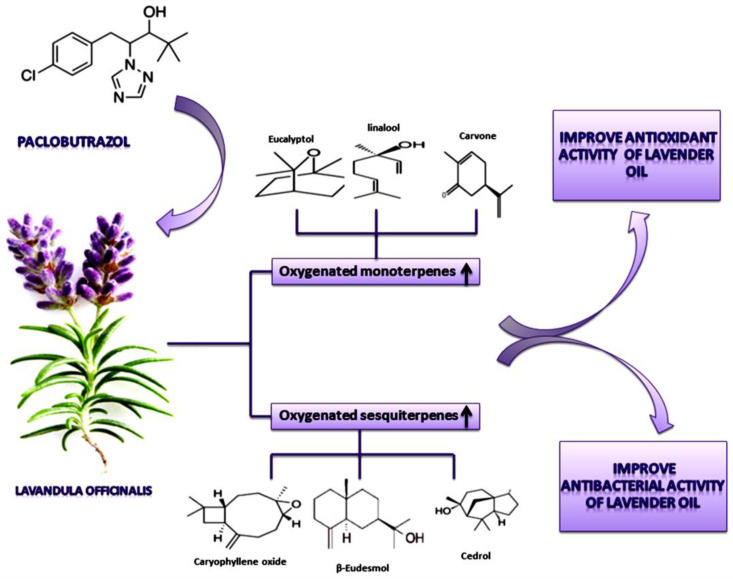
Simplified model for the suggested effect of paclobutrazol (PBZ), as a foliar application, on *Lavandula officinalis* L., improving the Antioxidant and Antimicrobial Properties of the extracted oil through modification of its composition from the oxygenated terpenes.

The chemical characterization of *Lavandula officinalis* L., as well as its antioxidant and antibacterial properties, were investigated in this study. It could be concluded that the essential oil of plants treated with 400 ppm PBZ was found to be very rich in oxygenated monoterpenes and oxygenated sesquiterpenes, which remain the main contributors to the biological activities of this oil. Our study showed that the essential oil extracted from plants, treated with 400 ppm PBZ, was more effective against the tested microbes than the other treatments, aside from its greater activity of antioxidant properties (Figure 8).

## Figures and Tables

**Table 1:** Quantity of monoterpene and sesquiterpene constituents of the lavender essential oil (%), identified by GC-MS analysis of untreated with paclobutrazole lavender plants (control).

	Compound	Control		PBZ Treatments		
No.				200 ppm	400 ppm	600 ppm
	**Monoterpene Hydrocarbons**					
1	α-Pinene		1.07	-	-	-
2	β-Pinene		0.95	-	0.69	-
3	3-Carene		0.38	-	0.84	0.22
4	γ -Terpinene		0.89	-	0.92	-
5	Terpinolene		0.91	-	-	-
	**Total (%)**		**4.2**	**-**	**2.45**	**0.22**
	**Oxygenated monoterpenes**					
1	Carveol		0.98	-	-	0.37
2	Eucalyptol		21.55	29.89	22.95	0.64
3	α-Terpineol		6.07	-	5.35	-
4	*p*-Cymen-7-ol		0.79	-	0.71	-
5	Linalool		-	-	2.25	-
6	L-Pinocarveol		-	-	1	-
7	α-Pinocarvone		1.52	-	1.65	-
8	cis-Verbenol		-	-	0.24	-
9	2-Pinen-10-ol		-	-	1.25	-
10	trans-Carveol		0.94	-	-	0.43
11	L-camphor		17.56	13.76	16.67	9.98
12	D-Carvone		-	-	1.93	-
	**Total (%)**		**49.41**	**43.65**	**54**	**11.42**
	**Sesquiterpene Hydrocarbons**					
1	β-Copaene		0.22	-	-	-
2	Caryophyllene		0.47	-	0.61	-
3	α-Farnesene		-	-	3.8	-
	**Total (%)**		**0.69**	**-**	**4.41**	**-**
	**Oxygenated sesquiterpenes**					
1	β-Spathulenol		0.69	-	0.43	-
2	Caryophyllene oxide		0.23	-	0.25	9.94
3	β-Eudesmol		-	-	1.28	-
4	Humulene oxide II		0.64	-	-	-
5	δ-Cedrol		-	-	3.2	-
6	Nootkatone		0.37	-	0.52	-
	**Total (%)**		**1.93**	**-**	**5.68**	**9.94**

## Data Availability

Not applicable.

## Supplementary Materials

The following are available online at https://www.mdpi.com/article/10.3390/plants11121607/s1, Table S1: Quantity of Constituents of the Lavender Essential Oil (%) Identified by GC-MS Analysis of Untreated and Treated with Paclobutrazole Lavender Plants.
