# Supplementary material for: Exogenous Paclobutrazol Reinforces the Antioxidant and Antimicrobial Properties of Lavender (Lavandula officinalis L.) Oil through Modulating Its Composition of Oxygenated Terpenes
Source: Plants (Basel). 2022 Jun 19;11(12):1607. doi: 10.3390/plants11121607 (PMC9230930; doi:10.3390/plants11121607)
Supplement: Supplementary file 1 [file plants-11-01607-s001.zip › plants-1780580-supplementary.pdf]

Table S1. Quantity of Constituents of the Lavender Essential Oil (%) Identified by GC-MS Analysis of Untreated and Treated with Paclobutrazole Lavender Plants

No.	Compound	Control	T1 (200ppm paclobutrazole)	T2 (400ppm paclobutrazole)	T3 (600ppm paclobutrazole)
2	α-Pinene	1.07	-	-	-
3	β-Pinene	0.95	-	0.69	-
4	Carveol	0.98	-	-	0.37
5	α-Phellandrene	2.01	-	0.66	-
6	Eucalyptol	21.55	29.89	22.95	0.64
7	γ -Terpinene	0.89	-	0.92	-
8	Terpinolene	0.91	-	-	-
9	L-camphor	17.56	13.76	16.67	9.98
10	α-Pinocarvone	1.52	-	1.65	-
11	α-Terpineol	6.07	-	5.35	-
12	Limonene dioxide	2.22	-	-	-
13	α-Terpinyl acetate	3.17	-	0.63	-
14	Myrtenol	2.55	-	-	-
16	trans-Carveol	0.94	-	-	0.43
17	Isocaryophyllene	2.22	-	-	-
18	Acorenone B	0.42	-	-	-
19	L-α-bornyl acetate	0.28	-	-	-
20	p-Cymen-7-ol	0.79	-	0.71	-
21	Hexyl tiglate	1.02	-	-	-
22	Lanceol, cis	0.39	-	0.25	-
23	Nootkatone	0.37	-	0.52	-
24	Caryophyllene oxide	0.23	-	0.25	9.94
25	β-Copaene	0.22	-	-	-
26	Viridiflorene	0.34	-	-	-
27	Thujopsen	0.49	-	-	-
28	Caryophyllene	0.47	-	0.61	-
29	β-Vetivenene	0.5	-	0.87	-
30	α-Copaen-11-ol	0.67	-	-	-
31	Cadinene	0.46	-	-	-
32	β-Eudesmene	0.4	-	-	-
33	β-Spathulenol	0.69	-	0.43	-
34	δ -EIemene	0.55	-	0.42	-
35	Valencene	2.87	-	-	-
36	trans-Calamenene	0.53	-	-	-
37	α-Calacorene	0.78	-	0.31	-
38	Humulene oxide II	0.64	-	-	-
39	Costol	0.41	-	-	-
40	β-Santalol	1.31	-	1.99	-
41	Ledol	0.52	-	0.74	-
42	Epicubebol	0.92	-	-	-
43	δ -Cadinol	0.57	-	-	-
44	α-epi-Muurolol	2.21	-	-	-

**45**	Elemol	0.78	-	-	-
**46**	Corymbolone	0.24	-	-	-
**47**	Clovane	2.09	-	-	-
**48**	Dehydroxy-isocalamendiol	0.46	-	-	-
**49**	Cedrenol	0.74	-	-	-
**50**	Longiverbenone	0.68	-	-	-
**51**	α-Santonin	0.45	-	-	1.34
**52**	γ -Elemene	0.51	-	-	-
**53**	Phytol	0.78	-	0.61	2.26
**54**	1-Octadecyne	0.44	-	-	-
**55**	Citronellyl valerate	0.56	-	-	-
**56**	Geranyl isovalerate	0.31	14.01	0.32	2.34
**57**	Kaempferitrin	0.56	-	-	-
**58**	Phytanic acid	0.31	0.63	-	2.14
**59**	Farnesol	0.26	-	0.36	-
**60**	1-Hexacosanol	0.26	-	-	-
**61**	2-Hexadecanol	0.4	-	-	0.27
**62**	Dihydrocarvone	-	0.37	3.6	1.29
**63**	Vitexin	-	1.93	-	2.15
**64**	3-(3,4-Dimethoxyphenyl)-4-methylcoumarin	-	4.92	-	5.32
**65**	2,4-Dimethoxy-2'-hydroxy-5'-methylchalcone	-	10.23	-	4.48
**66**	Gardenin	-	5.45	-	-
**67**	α-Fenchene	-	-	-	0.31
**68**	(+)-cis-Verbenol, acetate	-	-	-	0.33
**69**	β-Acorenol	-	-	-	.0.98
**70**	5β,7βH,10β-Eudesm-11-en-1α-ol	-	-	0.68	0.99
**71**	4-Hydroxy-b-ionone	-	-	-	3.47
**72**	5,8,11,14,17-Eicosapentaenoic acid, methyl ester, (all-Z)-	-	-	-	5.36
**73**	epi-g-Eudesmol	-	-	-	2.75
**74**	4-(3-Hydroxy-2,6,6-trimethylcyclohex-1-enyl)pent-3-en-2-one	-	-	-	4.18
**75**	n-Hexadecanoic acid	-	-	1.07	15.62
**76**	Hexa-hydro-farnesol	-	-	-	2.55
**77**	Farnesyl methyl ester	-	-	-	2.74
**78**	Isocalamenedio	-	-	0.34	2.67
**79**	6,7,3',4'-Tetramethoxyflavone	-	-	-	1.55
**80**	3',4',5',5,6,7-Hexamethoxyflavone	-	-	-	3.57
**81**	4',5,7-Trihydroxy3,6,8-	-	-	-	1.05

	trimethoxyflavone				
**82**	4,7-Dimethyl-3-(4-methoxyphenyl)coumarin	-	-	-	1.94
**83**	Pseudolimonen	-	-	1.73	-
**84**	1,3,8-p-Menthatriene	-	-	1.07	-
**85**	cis-Verbenol	-	-	0.24	-
**86**	Linalool	-	-	2.25	-
**87**	2-Pinen-10-ol	-	-	1.25	-
**88**	a-Farnesene	-	-	3.8	-
**89**	L-Pinocarveol	-	-	1	-
**90**	D-Carvone	-	-	1.93	-
**91**	Isoborneol, acetate	-	-	0.28	-
**92**	Piperitone	-	-	1.03	-
**93**	a-Gurjunene	-	-	0.33	-
**94**	a-Cedrene	-	-	0.44	-
**95**	Leden	-	-	3.58	-
**96**	Levorphanol	-	-	0.47	-
**97**	2,6,10-Dodecatrien-1-ol, 3,7,11-trimethyl	-	-	1.01	-
**98**	α-Santalol	-	-	0.42	-
**99**	Cubebol	-	-	1.2	-
**100**	3,4,5-Trimethoxycinnamic acid	-	-	0.23	-
**101**	d-Cedrol	-	-	3.2	-
**102**	b-Eudesmol	-	-	1.28	-
**103**	g-Himachalene	-	-	0.66	-
**104**	2H-Cycloprop[c]indene-2,3(3ah)-dione, hexahydro-3a,7,7-trimethyl	-	-	1.8	-
**105**	Torulosol	-	-	0.85	-
**106**	6,9,12-Octadecatrienoic acid	-	-	0.25	-
**107**	9,12-Octadecadienoic acid (Z,Z)-	-	-	0.79	-
**108**	Isophytol	-	-	0.27	-
**109**	Heptacosane	-	-	0.23	-
	Numberofdetected compound	61	9	54	31
